# mSphere of Influence: Evolutionary Strategies To Sensitize Drug-Resistant Pathogens

**DOI:** 10.1128/mSphere.00313-19

**Published:** 2019-06-26

**Authors:** Rebecca S. Shapiro

**Affiliations:** a Department of Molecular and Cellular Biology, University of Guelph, Guelph, Ontario, Canada

**Keywords:** antibiotics, antimicrobial drug resistance, collateral sensitivity, experimental evolution, microbial pathogens

## Abstract

Rebecca S. Shapiro studies antimicrobial resistance and genetic interaction networks. In this mSphere of Influence article, she reflects on how the papers “Bacterial evolution of antibiotic hypersensitivity” by Lázár et al. (V. Lázár, G. Pal Singh, R. Spohn, I. Nagy, et al., Mol Syst Biol 9:700, 2013, https://doi.org/10.1038/msb.2013.57) and “Use of collateral sensitivity networks to design drug cycling protocols that avoid resistance development” by L. Imamovic and M. O. A. Sommer (Sci Transl Med 5:204ra132, 2013, https://doi.org/10.1126/scitranslmed.3006609) impacted her thinking about multigene interaction effects on drug resistance.

## Abstract (precis)

Rebecca S. Shapiro studies antimicrobial resistance and genetic interaction networks. In this mSphere of Influence article, she reflects on how the papers “Bacterial evolution of antibiotic hypersensitivity” by Lázár et al. (V. Lázár, G. Pal Singh, R. Spohn, I.

## COMMENTARY

Early in my postdoctoral training, I was influenced by two complementary articles on antimicrobial collateral sensitivity, which exploited an understanding of drug resistance adaptation to devise novel strategies to manage antibiotic resistance: “Bacterial evolution of antibiotic hypersensitivity” by Lázár et al., in *Molecular Systems Biology* (1), and “Use of collateral sensitivity networks to design drug cycling protocols that avoid resistance development” by Imamovic and Sommer, in *Science Translational Medicine* (2), both published in 2013. These two articles sought to characterize antibiotic drug resistance profiles in a simple bacterial model and, moreover, to decipher complex collateral sensitivity networks between diverse antibiotics. Collateral sensitivity describes a phenomenon where the evolution of resistance to one drug consequently confers increased susceptibility to another drug—a sort of evolutionary tradeoff. Both these articles identify key collateral sensitivities between antibiotic agents; for example, they find that evolution of resistance to the aminoglycoside class of antibiotics often comes at the expense of increased sensitivity to other classes of antibiotic drugs, including quinolones and β-lactams. These studies elegantly demonstrate how such evolutionary tradeoffs can be exploited to increase antibiotic sensitivities within bacterial populations. Further, they suggest that collateral sensitivities could ultimately be exploited to inform treatment with combinations of antibiotics, to slow or prevent the emergence of drug resistance, and to enhance therapeutic efficacy.

For this research, both studies took an experimental evolution approach to drive the evolution of antibiotic drug resistance *in vitro*. Using Escherichia coli as a simple bacterial model system, both groups of researchers evolved dozens of lineages of bacteria in the presence of fixed or increasing concentrations of >20 distinct antibiotic drugs. The resultant experimentally evolved antibiotic-resistant populations were then assayed for resistance to panels of antibiotic drugs to which they had not previously been exposed, in order to identify novel collateral sensitivities. Both groups identified several instances of bacteria acquiring resistance to one antibiotic resulting in increased susceptibility to other antibiotics. In particular, collateral sensitivity between aminoglycoside and β-lactam antibiotics was identified by both groups. Imamovic and Sommer further demonstrated that cycling between these two classes of antibiotic drugs actively selects against antibiotic-resistant bacteria and effectively eradicates drug resistance *in vitro* (2). Lázár et al. used whole-genome sequencing to identify mutations associated with aminoglycoside resistance and suggested a putative mechanism whereby mutations affecting the proton-motive force across the bacterial inner membrane render cells hypersensitive to other classes of antibiotics (1).

While neither of these papers was the first to describe the phenomenon of collateral sensitivity (first described by Szybalski and Bryson [3]), they were the first to study this phenomenon in bacteria on a large scale and map out collateral sensitivity networks across dozens of distinct antibiotic drugs. Since my own research focuses on both antimicrobial resistance and genetic interaction networks, these papers influenced my thinking in several ways. First, I study genetic mechanisms of antimicrobial resistance, and I was enthusiastic to see this novel research that exploited the relatively simple idea of evolutionary tradeoffs to drive sensitivity or hypersensitivity to specific antibiotic drugs. That such naturally occurring tradeoffs could be coopted by researchers, or perhaps even clinicians, to actively select against antibiotic resistance is a very appealing concept, particularly in light of the growing crisis of drug-resistant infections. Second, another focus of my research is microbial genetic interactions (4) and elucidating how epistatic interactions between two mutant gene products can influence resistance to antimicrobial agents. The research presented in these two papers used a chemical-genetic approach to dissect how specific resistance mutations influence resistance or susceptibility to antibiotic drugs. I see these two approaches as highly complementary: collateral sensitivity profiling uses chemical-genetics to identify mutations that render cells hypersensitive to drugs, while genetic-interaction screening can identify secondary mutations (which could point to putative drug targets), which can sensitize drug-resistant mutants. These papers prompted me to consider how integrating these two approaches can advance our understanding of antimicrobial resistance and reveal novel strategies to promote antimicrobial susceptibility.

Since these papers were published, many more studies have delved further into the phenomenon of collateral sensitivity in diverse bacterial organisms. Recent work has revealed that collateral sensitivity networks are conserved in clinical isolates of E. coli and are generally predictable, based on the genetic mechanism of resistance (5). Collateral sensitivities have also recently been reported upon treatment with antimicrobial peptides (6), as well as antimicrobial natural products (7). Further, collateral sensitivities have been identified for other clinically relevant bacterial pathogens, including Pseudomonas aeruginosa (8, 9), and Staphylococcus aureus (10). While collateral sensitivity has also been described in cancer cell lines, as well in animal models of cancer (11, 12), it has yet to be comprehensively studied in eukaryotic microorganisms, including fungal pathogens and eukaryotic parasites, for which antimicrobial treatment options are limited and antimicrobial resistance is a growing concern. As such studies on collateral sensitivity progress, I am eager to learn if the rational design of treatment regimens based on collateral sensitivity profiles will have bearing in the context of complex clinical infections, and what additional challenges will remain in applying the theory learned in this research in clinical settings.
